# Distribution of the acoustic occurrence of dolphins during the summers 2011 to 2015 in the Upper Gulf of California, Mexico

**DOI:** 10.7717/peerj.9121

**Published:** 2020-05-20

**Authors:** Gustavo Cárdenas Hinojosa, Horacio de la Cueva, Tim Gerrodette, Armando M. Jaramillo-Legorreta

**Affiliations:** 1 Departamento de Biología de la Conservación, Centro de Investigación Científica y de Educación Superior de Ensenada, Ensenada, Baja California, México; 2 Comisión Nacional de Áreas Naturales Protegidas, Secretaría de Medio Ambiente y Recursos Naturales, Ensenada, Baja California, México; 3 Southwest Fisheries Science Center, National Oceanic and Atmospheric Administration, San Diego, CA, United States of America

**Keywords:** Vaquita refuge, Passive acoustic monitoring, Echolocation clicks, C-Pod, Generalized linear model, Natural protected areas management

## Abstract

Baseline knowledge of spatial and temporal distribution patterns is essential for cetacean management and conservation. Such knowledge is particularly important in areas where gillnet fishing occurs, as the Upper Gulf of California, which increases the probability of bycatch of cetaceans. In this area, the vaquita porpoise (*Phocoena sinus*) has been widely studied, but the knowledge of other cetaceans is scarce and based on traditional visual survey methods. We used data collected by an array of acoustic click detectors (C-PODs) during the summers 2011 to 2015 to analyze the distribution of dolphins in the Vaquita Refuge in the Upper Gulf of California. We recorded 120,038 echolocation click trains of dolphins during 12,371 days of recording effort at 46 sampling sites. Based on simultaneous visual and acoustic data, we estimated a false positive acoustic detection rate of 19.4%. Dolphin acoustic activity varied among sites, with higher activity in the east of the Vaquita Refuge. Acoustic activity was higher at night than during the day. We used negative binomial generalized linear models to study the count of clicks of dolphins in relation to spatial, temporal, physical, biological and anthropogenic explanatory variables. The best model selected for the response variable included sampling site, day-night condition, and vertical component of tide speed. Patterns in the spatial distribution of predicted acoustic activity of dolphins were similar to the acoustic activity observed per sampling season. Higher acoustic activity was predicted at night, but the tide speed variable was not relevant under this condition. Acoustic activity patterns could be related to the availability of prey resources since echolocation click trains are associated with foraging activities of dolphins. This is the first study of the distribution of dolphins in Mexico using medium-term systematic passive acoustic monitoring, and the results can contribute to better management to the natural protected area located in the Upper Gulf of California.

## Introduction

The Upper Gulf of California, Mexico, is one of the most biologically productive marine regions of the world (Brusca et al., 2017). This area is an important ground for artisanal fishing in Mexico (Rodríguez-Quiroz et al., 2010). Furthermore, industrial trawling and recreational sport fishing also occurs in the area (Cudney-Bueno & Turk-Boyer, 1998). Therefore, baseline knowledge of spatial and temporal patterns of distribution are important for management and conservation of cetaceans in this area. Intensive gillnet fishing (Rodríguez-Quiroz et al., 2012) and illegal fishing (CIRVA, 2016) overlap with the distributions of cetaceans, which increases the probability of bycatch mortality or alteration of habitat use.

The Upper Gulf of California is the habitat of the most endangered marine mammal of the world, the vaquita (*Phocoena sinus*) (Jaramillo-Legorreta et al., 2017). In this region two marine protected areas were established: The Upper Gulf of California and Colorado River Delta Biosphere Reserve (DOF, 1993) and the Vaquita Refuge (DOF, 2015). The Reserve was created to protect the ecosystems, the biodiversity, and species that are ecological and commercially important, endemic, or at risk of extinction. The Refuge was created specifically to conserve and protect the vaquita. Historically the vaquita population has declined because of unsustainable bycatch in gillnets (Jaramillo-Legorreta, 2008; Rojas-Bracho & Reeves, 2013). As part of the recovery plan for the vaquita, the Mexican Government implemented a passive acoustic monitoring program in the Vaquita Refuge to estimate population trend, and to improve or reinforce conservation actions (Rojas-Bracho et al., 2010). The experimental design of the monitoring program consists of 46 sampling sites to record echolocation clicks of vaquita with autonomous acoustic loggers known as C-PODs. This program started in 2011 and has documented the drastic decline of vaquitas because of bycatch in both legal fishing activities and, in recent years, intensive illegal fishing of totoaba (*Totoaba macdonaldi*) (Jaramillo-Legorreta et al., 2017; Thomas et al., 2017; Jaramillo-Legorreta et al., 2019).

The C-PODs are static passive acoustic monitoring loggers equipped with omni-directional hydrophones sensitive to record echolocation clicks from 20 to 160 kHz. The loggers can be used to monitor many odontocete vocalizations, such as narrow-band high-frequency (120–140 kHz) porpoise echolocation clicks and midfrequency (30–60 kHz) dolphin echolocation clicks. These devices also record boat sonar clicks (Tregenza et al., 2016). C-PODs have been used widely in ecological studies of porpoises and dolphins around the world, including studies of the interaction of harbor porpoises (*Phocoena phocoena*) and bottlenose dolphins (*Tursiops truncatus*) (Jacobson, Forney & Harvey, 2015), spatial and temporal variations in Indo-Pacific bottlenose dolphins (*Tursiops aduncus*) and the Indian Ocean humpback dolphins (S*ousa plumbea*) (Temple et al., 2016), Baltic harbour porpoise population distribution (Carlén et al., 2018), and studies with multiple dolphins species for categorizing or describing parameters of their click trains (Robbins et al., 2015; Palmer, Brookes & Rendell, 2017).

The literature about the distribution of dolphins in the northern Gulf of California is scarce. In this area the most commonly documented toothed cetaceans are bottlenose dolphins and long-beaked common dolphin (*Delphinus delphis bairdii*), although killer whales (*Orcinus orca*) have been recorded four times and false killer whales (*Pseudorca crassidens*) once (Silber et al., 1994; Henry et al., 2012; CIRVA, 2016). This work is the first report of acoustic monitoring of dolphins in the northern Gulf of California, with data collected each summer between 2011 and 2015. We describe diel patterns of acoustic activity and the distribution of dolphins based on echolocation click rates. This study provides solid baseline information for the future management and conservation plans of dolphins in the Upper Gulf of California.

## Material and Methods

### Data collection and processing

**Figure 1:**
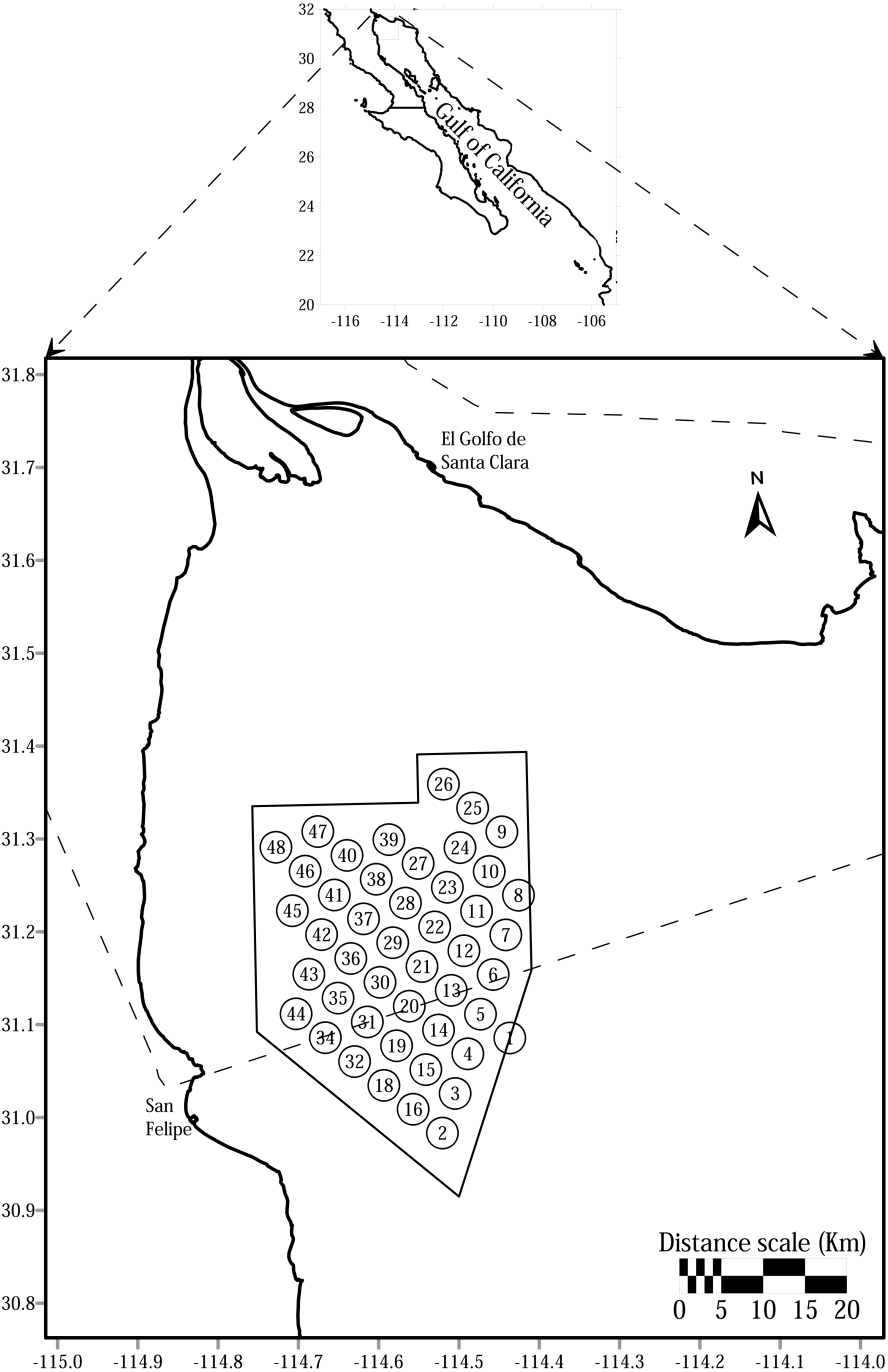
Sampling sites in the Vaquita Refuge located in the northern of Gulf of California, Mexico. The Refuge is limited with solid lines and the Upper Gulf of California with dash lines. The sites 17 and 33, initially located in the southwest border of the Vaquita Refuge, are not shown in the figure as they were removed from the monitoring program scheme because moorings deployed in these sites were lost all times in the first years of the study.

We used the dataset collected by the Program of Acoustic Monitoring of the Vaquita Population between the summers of 2011 to 2015. The data consist of a collection of clicks detected at a systematic array of 46 acoustic detectors, deployed during June to September each year, inside the Vaquita Refuge located in the western portion of the Upper Gulf of California, Mexico (Fig. 1). Based on the method applied by Jaramillo-Legorreta et al. (2017), in order to reduce bias we truncated the dataset to a core sampling period within which at least 50% of the detectors were operating across all 5 years. The core period goes from June 19th to August 19th. The total effort was calculated as the sum of days of recordings at each site during the five sampling seasons.

The autonomous acoustic logger used in this monitoring program was the C-POD (Chelonia Limited, UK. https://www.chelonia.co.uk). This device was designed to identify and store information of transient sounds (clicks) with fundamental frequencies between 20 and 160 kHz. Parameters used to describe clicks are dominant frequency, bandwidth, sound pressure level (peak-to-peak), duration (number of cycles), and time of detection (Tregenza et al., 2016). In contrast to storing complete waveforms, storing only parameters describing clicks reduces the load of memory needed, extending considerably sampling times. The C-POD uses a standard 4GB SD card.

A specialized program (CPOD.exe), provided by the manufacturer, was used to download data from SD cards, which produced CP1 files containing all the clicks identified. A proprietary algorithm (KERNO version 2.044), available in the same software, was used to classify click trains into four classes: “NBHF” (narrowband high-frequency click trains, typically emitted by porpoises), “Other cetaceans” (wideband click trains indicative of most dolphin species), “Sonar” (mostly sounds produced by echo sounders), and “Unclassed” (representing unclassified/unknown clicks) (Tregenza et al., 2016). The algorithm identifies coherent click trains based on sequences of similar regularly spaced clicks, based on a probabilistic model discarding trains that arose by chance (such as snapping shrimp or sediment transport sounds). The classifier also assigned a quality class (high, moderate, and low) indicating the strength of classification (Tregenza et al., 2016; Nuuttila et al., 2017).

The KERNO algorithm produces a CP3 file, with the same structure as CP1 files, but containing only the classified click trains. The click trains classified as NBHF in the acoustic monitoring of vaquita were manually inspected on screen and validated by analysts using the established acoustic parameters of vaquita (Jaramillo-Legorreta et al., 2017). During the inspection, the click trains classified as “Other cetaceans” were not validated as dolphins. These click trains are examined in this paper.

### Validation of dolphin click train identification

According to Palmer, Brookes & Rendell (2017), the use of C-POD data processed only with the KERNO algorithm is justified in studies where the researchers can be confident that most detections represent single dolphin and/or porpoise species. In our study, the two most common dolphin species were treated as a single category, and the vaquita is the only odontocete in the area capable of producing “NBHF” clicks.

We tested the validity of “Other cetaceans” click trains as a reliable identifier of dolphin acoustic activity by constructing a signature of dolphin click trains with acoustic data collected during sightings in the field. Between August 2013 to March 2014, we conducted six surveys to locate groups of dolphins in the same area where the main dataset was collected (Fig. 1). We collected the data under permits SGPA/DGVS/06998/11, and SGPA/DGBS/07105/12 of the Dirección General de Vida Silvestre, Secretaría de Medio Ambiente y Recursos Naturales, Mexico. The search effort was conducted in a non-systematic manner. We encountered long-beaked common dolphins and bottlenose dolphins. When a group of dolphins was sighted, the boat was moved in front of the group to deploy a floating mooring array of 3 C-PODs at depths of 5, 10, and 15 m. We tried to deploy the mooring at distances between 500 to 700 m in front of the group. However, it was common that groups changed their swimming trajectories before reaching the point of deployment. The alternative approach was to deploy three moorings with one C-POD at 10 m depth each, or two moorings with C-PODs at 5 and 10 m each, trying to position at least one in the trajectory of the groups.

During some sightings, we were able to retrieve and redeploy the moorings to collect more data. Once the moorings were deployed, the boat engine was turned off. All groups of dolphins sighted were composed of only one of the species. Finally, during the sightings, we recorded GPS locations, and times of deployment and retrieval of moorings. Behavior, group size, and the approximate distance of the animals to the moorings were recorded every five minutes.

Data were processed routinely to obtain CP1 and CP3 files. Similar to other studies, only high and moderate quality other cetaceans click trains were used in the analysis (Robbins et al., 2015; Palmer, Brookes & Rendell, 2017). Data of all click trains of interest, occurring during times of sightings, were characterized based on averages and standard deviations of click parameters, including only trains with at least five clicks. Additionally, the inter-click interval was used as a characterizing parameter (time between consecutive clicks in a train). For every parameter a signature interval included the mean and plus/minus one standard deviation. Due to the small size of recordings of bottlenose dolphins and high overlapping of the values of some acoustic parameters, data of both species, the bottlenose and the long-beaked common dolphins, were treated as a single statistical population of “dolphins” to construct the click train signature, assuming no false positive click trains occurred during sightings.

We developed an algorithm in MatLab (MathWorks trademark, R2015a) to test the signature against the “Other cetaceans” click trains classified by KERNO. The algorithm calculated means of parameters of trains classified as “Other cetaceans” in the dataset being tested, to compare them with the corresponding signature interval. If the mean of just one parameter lay out of the interval, the train was classified as false (Supplemental Information 1). First, we tested the signature against the data used to construct it. Finally, we applied the algorithm to the dataset of the core period collected between 2011 and 2015 to estimate the dolphin false positive rate of the click trains classified as “Other cetaceans” by KERNO. Therefore, the validity of KERNO to identify dolphin clicks was assessed based on the rate of false positives after applying the signature algorithm.

### Analysis of the distribution of the acoustic occurrence of dolphins

Acoustic detection rates were measured using click counts, irrespective of the number of trains detected. Trains included in the analysis had at least 5 clicks. We used average clicks per day per sampling site to describe the distribution patterns of the dolphins among the sampling seasons. We characterized the diel patterns of echolocation acoustic activity of dolphins using the mean of clicks per hour of the day. To examine the effects of light conditions on dolphin echolocation, each sampling day was divided into daylight and night periods established through sunrise and sunset times (rounded to the nearest hour) for the location of San Felipe obtained with the software Mar v10 (http://predmar.cicese.mx/). From June 19th to August 19th the average sunrise time for the sampling periods from 2011 to 2015 was 04:50:18 and the average sunset time was 18:39:21.

The clicks per hour were not normally distributed (Lilliefors, *p* < 0.05). Therefore, the differences of this variable among sampling seasons, time of day, and between daylight and night conditions, were tested with Kruskal–Wallis one-way analysis of variance. We used the dunnTest function in R, with Bonferroni correction, to apply a posteriori Dunn multiple comparisons test of means (Zar, 1999). In all analyses a *p* value < 0.05 was considered significant.

We used generalized linear models (GLM) to describe the acoustic occurrence of dolphins in relation to spatial, temporal, oceanographic, anthropogenic, and biological variables. The explanatory spatial variables were sampling sites and their location (latitude and longitude, UTM coordinate). To try avoiding biases, we removed data of samples sites number 12 and 18 of this analysis since no data were available at these sites in at least three field seasons because of loss of moorings with C-PODs (Supplemental Information 2). The temporal explanatory variables were the sampling season, time of day, and day/night period; the oceanographic variables were the temperature at 10 m below sea surface recorded by a sensor of the C-PODs, and the vertical speed component of the tide. This last variable was calculated by time of day averaging the difference of the tide heights every five minutes during the study period. The acoustic presence of vaquitas was the only biological variable considered in the analysis. Furthermore, boat sonar clicks were included as the anthropogenic explanatory variable. The response variable was the count of the echolocation clicks of dolphins per hour (Table 1). Therefore, all the explanatory variables of the database were calculated or arranged at periods of one hour (Supplemental Information 3).

**Table 1:** Description and type of the variables used for analyzing the acoustic occurrence of dolphins in the Refuge of Vaquita.

**Variable**	**Description**	**Type**
Dolphin clicks	Count of clicks of dolphins in hour period	Continuous
Site	Sampling site label	Categorical
Site locations	UTM locations (northing and easting)	Continuous
Day-night time	Daytime and nighttime condition	Categorical
Time of day	Time of day (rounded to hour)	Categorical
Month	Month of sampling period	Categorical
Sampling season	Year of sampling season	Categorical
Vaquita clicks	Count of clicks of vaquitas in hour period	Discrete
Sonar clicks	Count of clicks of sonar boats in hour period	Discrete
Depth	Depth of sampling sites (m)	Continuous
Sea temperature	Mean hour temperature at 10 m below the surface (° C)	Continuous
Tide	Vertical speed component of tide in hour scale (m/s)	Continuous

We searched for outliers and correlations between explanatory variables through Pearson correlation coefficient analysis. We used the software R (R Core Team, 2018) and the glm() stats package (Chambers & Hastie, 1992) to fit the GLM model. We specified a negative binomial family with a logistic link function using the glm.nb() function of the MASS package (Venables & Ripley, 2002). The best model (combination of explanatory variables) was selected using Akaike’s Information Criterion (AIC). To analyze the acoustic occurrence modeled per sampling season, we divided the database per season, and we calculated the predictions of click counts of dolphins per hour of the best model using the predict() function of the car package (Fox & Weisberg, 2019). Predicted click rates at each sampling site per sampling season were calculated to visualize the spatial patterns modeled for dolphin activity. The scale of the predicted click rates was changed to day-scale for comparative purposes with the observed data (clicks/day).

## Results

### Data

During the 2011–2015 sampling seasons of the acoustic monitoring program, we collected 12,371 days of recording effort from June 19th to August 19th of each year in a total of 214 sites sampled (Table 2). A total of 1,647,415 clicks from 120,038 trains were classified as dolphins by the KERNO algorithm, with 21.17% (25,417) of the trains labeled as “high” quality, and 78.82% (94,621) as “moderate” quality (Table 2). Vaquita and sonar clicks were also recorded and used in the GLM analysis.

**Table 2:** Summary of effort and of acoustic data of dolphins, vaquitas and sonar recorded from June 19th to August 19th during 2011 to 2015 sampling seasons.

	**2011**	**2012**	**2013**	**2014**	**2015**	**Total**
Sampling sites	40	45	43	40	46	214
Days recorded	2,280	2,714	2,299	2,452	2,626	12,371
Dolphin clicks	464,511	112,382	244,444	466,655	359,423	1,647,415
Dolphin click trains	36,759	8,727	16,801	34,222	23,529	120,038
High quality clicks trains dolphins	7,116	7,553	2,093	4,593	4,062	25,417
Moderate quality clicks trains dolphins	29,643	1,174	14,708	29,629	19,467	94,621
Vaquita clicks	363,938	423,911	238,624	136,033	91,057	1,253,563
Vaquita click trains	31,027	34,912	19,801	11,404	7,699	104,843
Sonar clicks	11,303	949,468	6,795	39,529	132,433	1,139,528

During the surveys to collect echolocation clicks of dolphins, we recorded 14 sightings of long-beaked common dolphins and three of bottlenose dolphins. Mean group size was 246 (SD = 315) for common dolphins and 9 (SD = 4) for bottlenose dolphins. Over all surveys, we deployed C-PODs during 8.18 h of effort, and we recorded 3.6 min of click trains. The distance of the animals to the loggers ranged from 0 to approximately 1,000 m. A total of 12,490 clicks from 837 click trains of dolphins were recorded, with a range of 5 to 89 clicks from each train. Of the click trains, 36% (301) were labeled as “high “quality and 64% (536) as “moderate” quality. Most of the total clicks were logged for the long-beaked common dolphin with 12,215 clicks distributed in 823 trains. Only 275 clicks belonging to 14 click trains were logged for the bottlenose dolphin. However, we only spent 22.19% of the total time of the recording effort with this species and its group size was small.

Mean or standard deviation values of the two species showed an overlap or similarity in some dolphin click train parameters (Table 3). The average of the number of cycles (duration) was almost the same for both species. The mean of the minimum frequency of common dolphins was higher than bottlenose dolphins, but the means of maximum, modal and mean frequencies were lower. Also, the interclick intervals and duration time of the click trains were higher for bottlenose dolphins. Clicks per second of common dolphins was the only acoustic parameter with a notably higher value than for bottlenose dolphins.

**Table 3:** Data of acoustic features of bottlenose dolphins, long-beaked common dolphins, and both species pooled (Dolphins), based in the click trains collected during sightings in the Vaquita Refuge. All the values are mean and in parenthesis is the standard deviation value. Asterisks show the variables selected to construct the signature for dolphins.

**Parameters**	***D. d. bairdii*** ***N* = 823**	***T. truncatus*** ***N* = 14**	**Dolphins** ***N* = 837**
Frequency (kHz)	62.91 (±13.95)	88.43 (±19.19)	63.69 (±14.78)
Modal Frequency (kHz)^*^	71.77 (±16.54)	93.92 (±34.85)	72.14 (±17.21)
Minimum Frequency (kHz)	62.01 (±16.66)	49.14 (±20.42)	61.8 (±16.68)
Maximum Frequency (kHz)	82.48 (±21.79)	123.42 (±26.13)	73.16 (±22.48)
Interclick intervals (ms)	20.27 (±40.25)	77.33 (±73.49)	22.0 (±42.68)
Click train duration time (ms)	235,37 (±752.06)	1,494.24 (±1,877.55)	256.434 (±798,14)
Click duration (number of cycles)^*^	5.73 (±1.22)	5.57 (±1.08)	5.72 (±1.21)
Clicks per second	348.49 (±304.93)	71.78 (±137.93)	343.86 (±304.92)

However, despite the differences in some acoustic parameters, for the purpose of this study both species were treated as a single population because of the small sample size for bottlenose dolphins. Therefore, we used the average of the acoustic characteristics of the two species to construct a signature for both species (labeled as dolphins) and to evaluate the accuracy of the routine and the false positive detections of dolphins in the data of the acoustic monitoring program of vaquita (Table 2).

### Validation of dolphin click train identification

We selected duration and modal frequency as the criteria parameters for our algorithm (Supplemental Information 1). We selected duration because of the similarity of the mean for the two species of dolphins (5.7 cycles for common dolphins and 5.5 cycles for bottlenose dolphins). The frequency (kHz) is a parameter widely used for classification of click trains of toothed whales. Since frequency had a lognormal distribution, we selected the modal frequency, since this central tendency measure is not affected by the tail of the distribution of the data when the sample size is large.

The values of the first and last quartiles used to characterize the signature of dolphins were 4 and 8.5 for click duration and 44 and 111 kHz for modal frequency. A train was rejected when any of the parameters lay outside of the corresponding interval (algorithm in Supplemental Information 1).

The algorithm estimated a rate of 5.9% of false negatives in the dataset of the dolphin click trains collected during the field surveys. Therefore, the algorithm performance was 94.1% accurate.

In the full dataset of the acoustic monitoring program, the algorithm estimated a rate of 19.4% false positive click trains classified as dolphins.

### Diel patterns

We found significant differences in the acoustic activity of dolphins between day and night (Kruskal–Wallis, *p* < 0.05). The overall mean dolphin click was 9.8 clicks/hour (SD = 143.4) during the night, and 2.1 clicks/hour (SD = 57.2) during the day.

**Figure 2:**
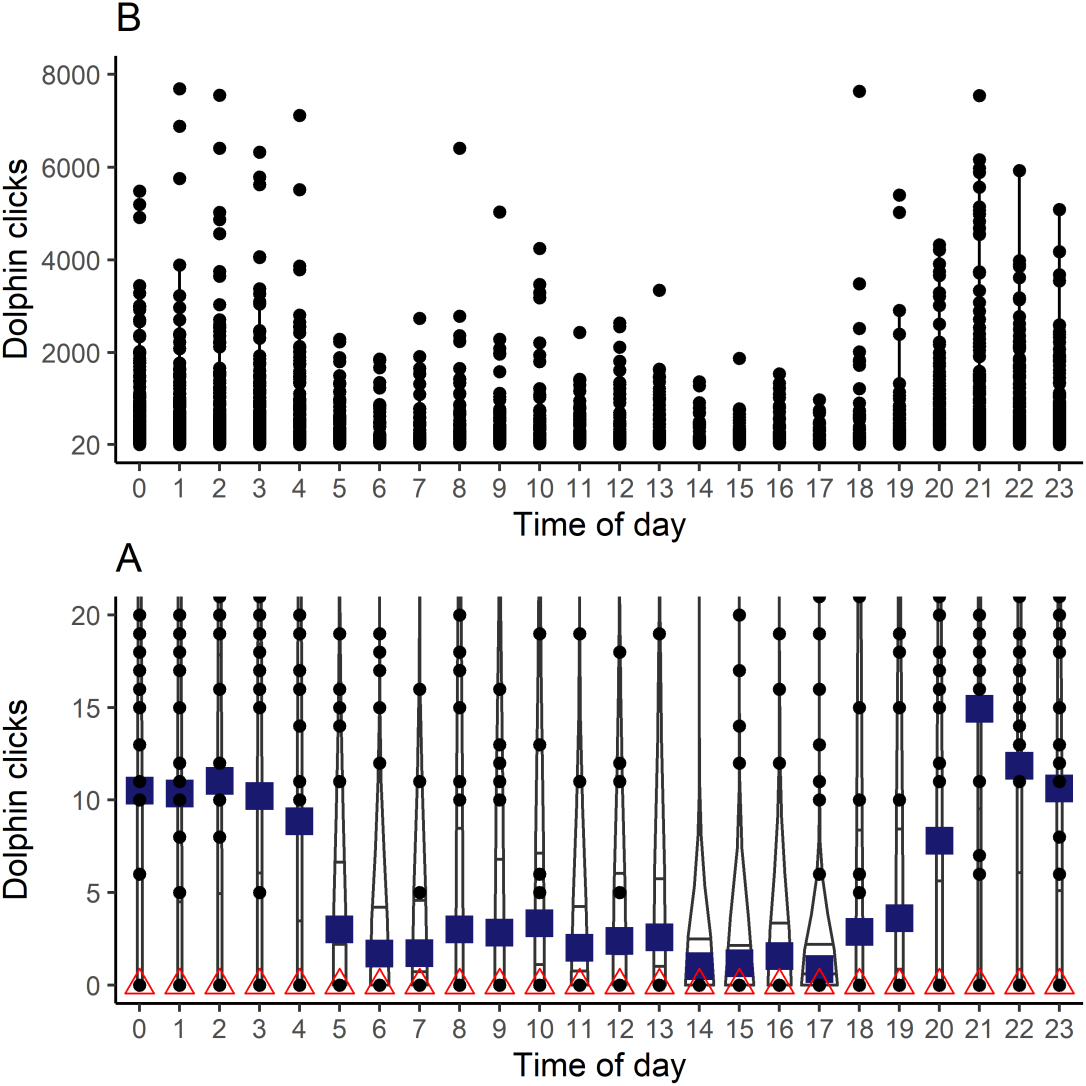
Distribution of clicks count recorded of dolphins by the time of day during the period study. (A) shows data with a range of y axis from 0 to 20 clicks. (B) shows data with a range of y axis from 20 to 8,000 clicks. The blue square indicates the mean and the red triangle the median.

Mean clicks per hour showed a clear diel pattern with a peak at 21:00 h (Fig. 2). Because of the excess of zeros in the counts of clicks per hour, the median clicks per hour were all zeros. The Kruskal-Wallis test indicated that the differences in acoustic activity per hour of the day were significant (*p* < 0.05). The Dunn’s test showed significant differences in the time ranges from 2000 to 0300 h and from 0400 to 1900 h (*p* < 0.05). Overall mean dolphin click rate was 10.9 clicks/hour (SD = 144.6) for the time ranges from 2000 to 0300, and 2.65 clicks/hour (SD = 61.7) from 0400 to 1900 h (Fig. 2). The latter was the range with most of the hours with daylight according to the mean sunset and sunrise times average for the study period.

### Spatial and annual patterns

**Figure 3:**
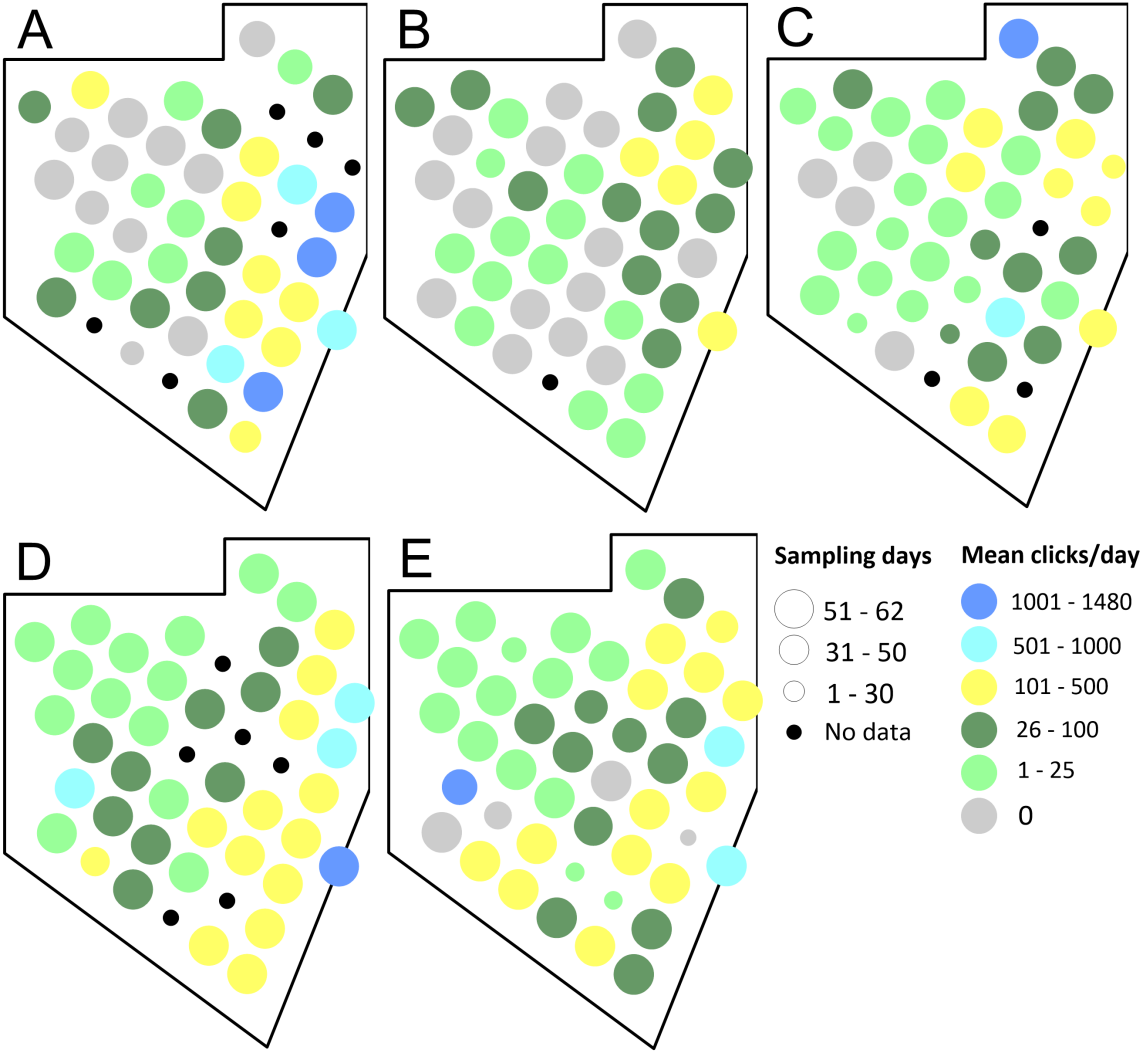
Spatial distribution of the observed acoustic occurrence of the dolphins in the Vaquita Refuge during the 2011–2015 sampling seasons. Sampling seasons: (A) 2011, (B) 2012, (C) 2013, (D) 2014, and (E) 2015. The black circles indicate the sampling site was missed in each sampling season. The size of the circles indicates the number of sampling days during each year.

The spatial pattern of click rates varied among years (Fig. 3). During the 2011 sampling season, there was low acoustic activity (0 to 25 clicks/day) mainly in the northwestern corner of the Vaquita Refuge, while in 2012 low activity was distributed almost homogeneously among the sampling sites. During 2013, 2014, and 2015 seasons the lowest values were located mainly in the northwestern corner of the study area. In general, in the 2011–2015 field seasons the sites with an average of clicks greater than 100 clicks per season were concentrated mainly along the eastern border of the Refuge. From 2011 to 2014, no data were available for 16 sampling sites because the moorings or CPODs were stolen or vandalized. Only during 2015, no loss of CPODs occurred (details in Supplemental Information 2).

The overall mean of the echolocation click rate per day for all the sampling seasons was 5.4 (SD = 103.12). Mean click rates per day for each season were 8.08 for 2011, 1.72 for 2012, 4.43 for 2013, 7.84 for 2014, and 5.33 clicks/season for 2015. The annual rates were statistically different (*p* < 0.05) among each pair of years (Dunn’s test, *p* < 0.05), except the comparison between 2013 and 2015.

### Model of the acoustic occurrence of dolphins

#### Data exploration

Data were visually inspected through the construction of boxplots. We found 99% of zeros in the response variable dolphin clicks per hour. In this variable, we also found five extreme values (>8,000 clicks/hour) and these outliers were eliminated from the database. With the number of zeroes found, we chose to model the counts of dolphin clicks in relation to the explanatory variables with a binomial negative family distribution. Before applying the generalized linear model, we also explored collinearity of the explanatory variables in order to avoid redundant information about the response. The Pearson correlation coefficient between UTM east and depth was high (0.69) and statistically significant (*p* < 0.05), and therefore we chose to eliminate the UTM east variable as an explanatory variable.

#### Results of the GLM models

**Figure 4:**
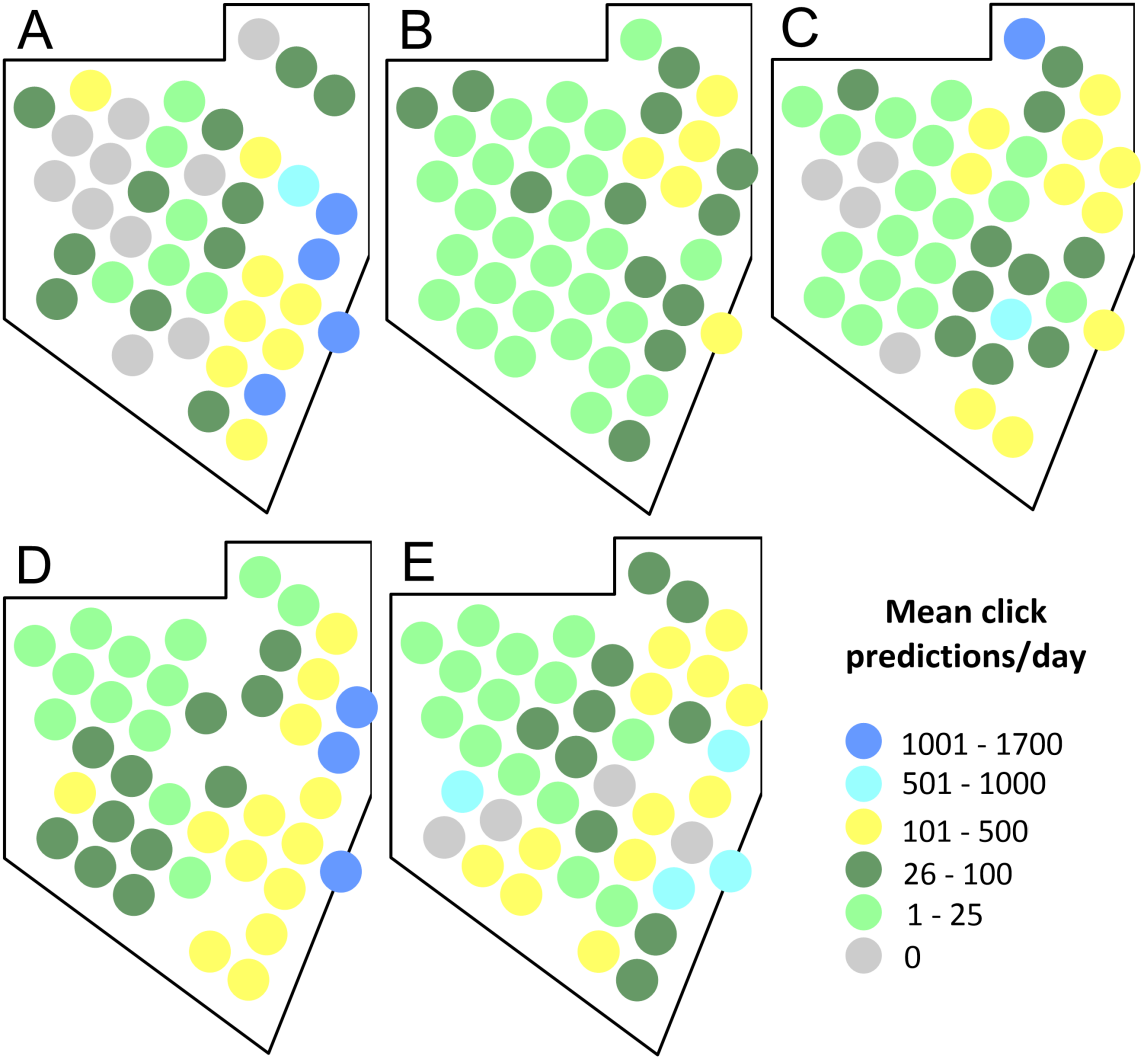
Spatial distribution of the predicted acoustic occurrence of the dolphins in the Vaquita Refuge during the 2011–2015 sampling seasons. Sampling seasons: (A) 2011, (B) 2012, (C) 2013, (D) 2014, and (E) 2015. Sampling sites with no data available in at least three field seasons were not included in the GLM analysis.

**Figure 5:**
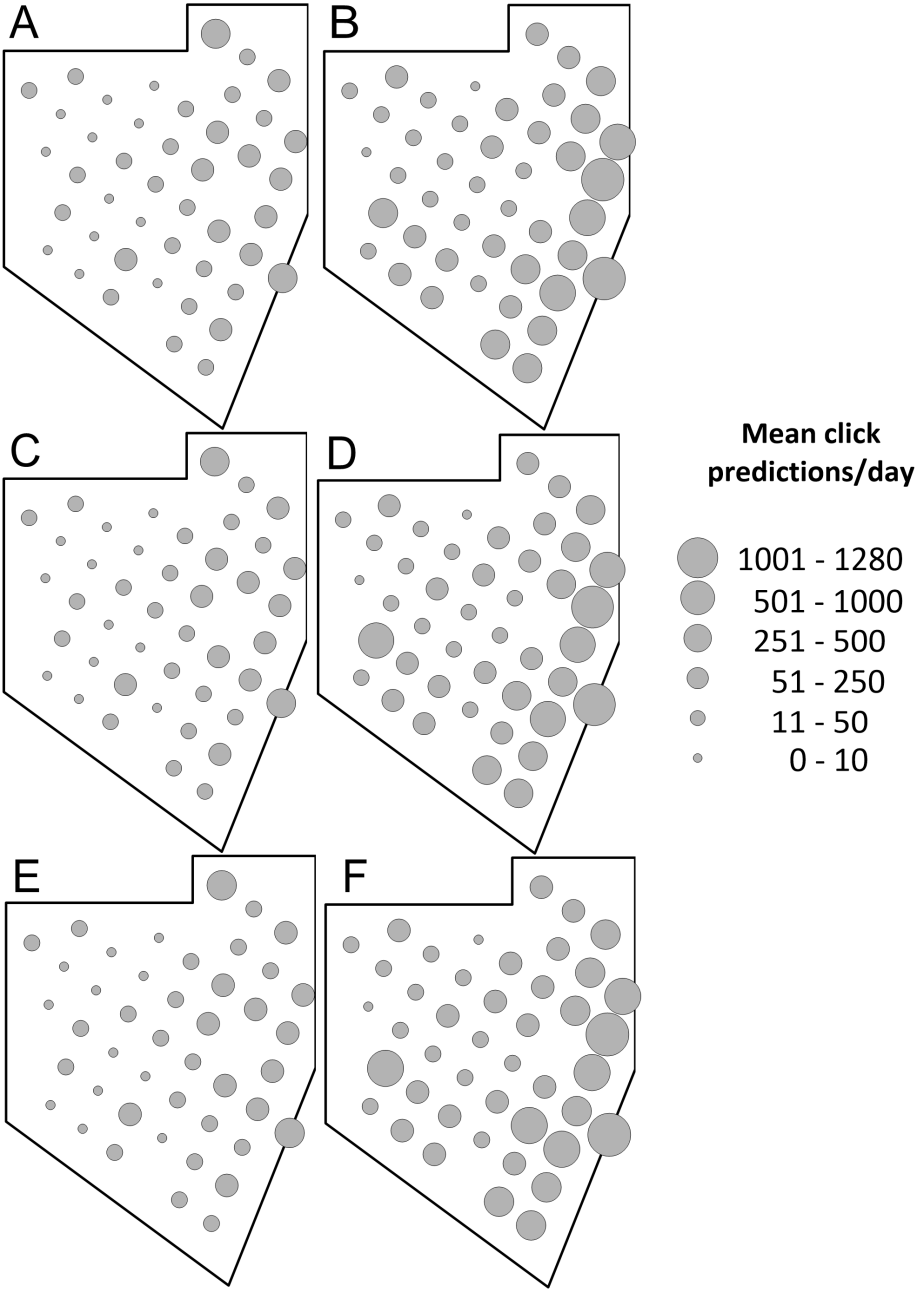
Dolphin mean clicks predictions rate visualized by the light condition of the day and by vertical speed component of the tide resulted from the best GLM model. Mean click predictions per (A) day and high-speed tide, (B) night and high-speed tide, (C) day and moderate-speed tide, (D) night and moderate-speed tide, (E) day and low-speed tide, and (F) night and low-speed tide.

Our formulation of combinations of variables resulted in 26 generalized linear models (Supplemental Information 4). Fourteen of these models converged. Table 4 shows the AIC values of these models. The model with the lowest AIC (73,889) included the interaction of the sampling site and day-night condition variables and the tide speed variable. Another model with sampling site and day-night interaction plus tide speed and depth interaction showed a very similar AIC value (73,891). However, we selected a formulation with fewer variables to obtain the simplest model for the counts of echolocation clicks of dolphins. The models which only included spatial (sampling site), temporal, biological, oceanographic, daylight-night condition or anthropogenic variables performed poorly, indicating that none of these models alone are adequate to explain the dolphin acoustic activity (Table 4). All the models with lower values of AIC included sampling site and the day-night conditions, indicating that these two variables are key for understanding the distribution of dolphin acoustic activity. The acoustic activity of dolphins predicted by the best model and the observed mean click rates over the five sampling seasons had similar spatial distributions, with the most of higher values in the eastern side of the Vaquita Refuge (Fig. 4), indicating a reliable fit of the model to the data. We also graphically analyzed the effect of the vertical tide speed component by using three categories: low (0–0.005 m/s), moderate (0.005–0.015 m/s), and high(0.015–0.03 m/s) vertical tide speed, separately by daylight condition (day vs night). This showed us that the higher echolocation activity (clicks predicted by day) of dolphins was located mainly in the eastern sampling sites and was determined by the night condition irrespective of the tide (Fig. 5). Furthermore, the predicted dolphin click rates output for the low, moderate, and high vertical speed of the tide were very similar during the day condition with the higher click rates distributed in the east of the Vaquita Refuge like in the night condition (Fig. 5).

**Table 4:** Formulation of the variables of the generalized linear models of the count of dolphin echolocation clicks. The AIC values are shown for each model. Interactions terms are shown with a ‘×’ between variables. The asterisk indicates the best model selected.

**Models.** **Response variable:** Counts of dolphin clicks/hour	**AIC**
Site × Day-night + Tide *	73889
Site × Day-night + Tide × Depth	73891
Site + Time of day + Tide	73952
Site + Time of day + Depth	73694
Site + Time of day + Depth + Day-night	73694
Day-night + Vaquita + Tide + Site	73966
Site + Day-night	73982
Site + Day-night + Tide	74210
Site	74225
Only temporal variables	74381
All biological and oceanographic variables	74486
Day-night × Tide	74531
Day-night condition	74540
Anthropogenic variable (sonar clicks)	74677

## Discussion

Passive acoustic monitoring is a method of increasing importance for the study of the distribution and occurrence of cetaceans. In the Upper Gulf of California, Mexico, dolphin acoustic activity varied among sites, with higher activity in the eastern part of the Vaquita Refuge. Acoustic activity was higher at night than during the day. The best GLM model to predict dolphin acoustic activity included sampling site, day-night condition, and vertical component of tide speed variables. The spatial distribution of predicted acoustic activity showed a pattern similar to the distribution of acoustic activity per sampling season. The higher values predicted occurred during the night condition, and the tide speed variable was not relevant for the acoustic activity of the dolphins during the night. The acoustic activity patterns obtained could be related to the availability of prey resources, since echolocation click trains are associated with foraging activities by dolphins. This is the first study of the distribution of dolphins in Mexico using medium-term systematic passive acoustic monitoring, and the results can contribute to better management of the natural protected area located in the Upper Gulf of California.

### Limitations in detection of echolocation click trains of dolphins

Our results based on the echolocation click trains of dolphins have some limitations. For example, according to Au (1993), bottlenose dolphins use echolocation mainly when feeding or travelling but less when socializing and resting. Furthermore, since the echolocation signals of dolphins are highly directional, only those vocalizations directed towards a logger will have a chance of being detected (Nuuttila et al., 2017). During our field surveys, we probably witnessed these limitations. Several times, when we deployed C-POD arrays in front of traveling groups of dolphins, we did not record a single click train, even though the animals swam directly past the array. In contrast, when we deployed C-POD array near groups of dolphins (bottlenose and common dolphins) feeding at the surface, we were able to record echolocation click trains.

Another limitation is that the high frequency signals of toothed whales attenuate quickly, and, therefore, they are detectable at most a few hundred meters away from the device (Philpott et al., 2007; Rayment, Dawson & Slooten, 2009). A study in Cardigan Bay using C-PODs reported a maximum detection distance ranging from 1,343 to 1,749 m for bottlenose dolphins (Nuuttila et al., 2013). In an assessment of C-POD performance in New River, North Carolina, the echolocation click trains of bottlenose dolphins were detected at a maximum distance of 933 m (Roberts & Read, 2015). We did not find records in the literature of C-POD distance detection ranges for common dolphins, but it is likely to be less than the 4,500 m between our sampling sites. For both dolphin species, therefore, there were large spaces between the C-PODs where acoustic activity could not be detected.

A further limitation was that the acoustic activity of the two dolphin species could not be separated. We believe that most of the recorded clicks in our study were due to long-beaked common dolphins rather than bottlenose dolphins, for two reasons. First, long-beaked common dolphins were more frequently encountered than bottlenose dolphins during our sampling surveys in the Vaquita Refuge. More extensive visual surveys carried out between February and May of 1986 to 1988 also reported that common dolphins were the most frequently encountered cetaceans in the Northern Gulf of California more than 11 km from shore (Silber et al., 1994), which is the area where C-PODs were located. Second, the larger group sizes of common dolphins increase the probability of recording echolocation clicks when a foraging group is near a recording device. Both the limited surveys of this study and the larger surveys of Silber et al. (1994) found that the average group size was about 250 for common dolphins and about 10 for bottlenose dolphins.

Finally, it is important to mention that, like the studies of vaquitas done by Taylor et al. (2017) and Thomas et al. (2017), we assumed that there are no reasons to expect systematic changes in click source level, click propagation, or background noise among years that could affect the detection of echolocation click trains of dolphins, and that factors that may affect acoustic behavior of dolphins, such as moon phase or tide cycles, were balanced between years because sampling was conducted during the same 62 calendar days each year.

### Validation of dolphin echolocation click trains

Instead of inspecting visually a random sample of click trains to validate the classification of KERNO algorithm and estimate the false positive rate that way, we chose to develop a simple algorithm whose performance was acceptable. The notable difference of the mean modal frequency and/or the duration of click trains of dolphins (72 kHz & 5.72 cycles) with other sounds sources as vaquita (128–139 kHz & >10 cycles), and sonar (50 kHz & >20 cycles) were key also to constructing a signature that distinguished dolphins from vaquitas or sonar. Cycles duration average values were similar to the values obtained for common dolphins (6.0 cycles) and bottlenose dolphins (5.27 cycles) in Broadhaven Bay, Ireland (Robbins et al., 2015). Furthermore, the selection of high and moderate quality click trains of “Other cetaceans” KERNO classifications probably increased the chance that our algorithm avoided false positives.

However, it is possible that the performance of the KERNO algorithm could be different depending on background noise specific to each sampling site and therefore possibly affected the performance of our algorithm. According to Nuuttila et al. (2013), a low false positive rate of click trains is expected when the background noise is low and even “low quality” class (assigned by KERNO) trains have a high chance to be true positive dolphin signals. The same authors suggested that interference from other sound sources can also affect the performance of the click train detection. In the study of Robbins et al. (2015), they found that the accuracy of the classifiers and quality classes of KERNO detections were site-specific. In our study area background noise is probably high due to different sources, such as biological factors (presence of snapping shrimps, fish, etc.), fishing boats, and mass transport of suspended sediments in the Upper Gulf of California due to extreme tidal ranges.

However, we were able to opportunistically test the performance of C-PODs during noisy conditions due to anthropogenic sources. We recorded 65 click trains of dolphins during one encounter of two groups of common dolphins (20 and 40 approx. group sizes) following two shrimp trawlers for foraging potentially of the bycatch discarded by fishermen. Other studies have also documented low false positive rates of bottlenose dolphin click trains for KERNO performance. In New River, North Carolina, Roberts & Read (2015) found that the C-PODs reported only a small number of false detections, as indicated by low false positive rates ranging between 1% and 4% for individual units, and in overall KERNO performed with high accuracy (72%–91%). Low false positives rates have been also found for Hector’s dolphins (Rayment, Dawson & Slooten, 2009) and harbor porpoises (Kyhn et al., 2012). The study of Robbins et al. (2015) developed acoustic parameters based on click trains of three species of dolphins (the bottlenose, common, and Risso’s dolphins (*Grampus griseus*)) for verification of dolphin detections from C-PODs. The authors reported a 68% false positive rate for the three quality classes (high, moderate, and low) of dolphin KERNO detections.

In our study, a limitation of our dolphin signature was the small sample size of dolphin click trains collected during the sightings. Range values of the dolphin signature (modal frequency and duration) could be biased because we may not have collected enough data on click trains for traveling or for foraging behaviors. Furthermore, our signature was based mainly on common dolphins, and this potentially overestimated our false positive rate, since we do not know, due to limited studies, if bottlenose dolphins are more abundant than common dolphins during summer in the Vaquita Refuge. Despite limitations, we suggest our positive false rate of dolphins was moderate (19.4%) in comparison to other studies, and we assumed our results based in the click trains of dolphins classified by KERNO are reliable to infer the distribution of the echolocation acoustic activity of dolphins.

### Distribution of the occurrence of echolocation clicks of dolphins

The higher acoustic activity of dolphins at night found in this study is in general agreement with other studies where the echolocation clicks of dolphins have been analyzed. Tregenza et al. (2016) mentioned that strong diel, tidal and seasonal patterning of the animals’ habitat use has been found including coastal sites that are regularly used only at night. In a study of bottlenose dolphins in Doubtful Sound, New Zealand, buzzes and echolocation activity in general were both significantly more common during dawn and dusk, suggesting crepuscular foraging (Elliott, Dawson & Henderson, 2011). In seven Spanish Mediterranean Marine Protected Areas a general observation across all the sampling seasons of the study was the preference by dolphins for night-time periods (Castellote et al., 2015). In another study using C-PODs in Menai Bay, Tanzania, the diel cycle also was the most significant temporal variable influencing occurrence of dolphins at sampling sites, with a probability of occurrence at night significantly higher than during daylight, sunrise, and sunset (Temple et al., 2016).

Bottlenose dolphins produce echolocation click trains mainly during feeding or travelling but less so during socializing and resting (Au, 1993). Vocalizations of the common dolphins in the Southern California Bight have a diel pattern, since the foraging behavior mainly occurred at night and travel and social behavior occurred during the day (Henderson et al., 2012; Wiggins et al., 2013). Therefore, our results about the distribution of echolocation activity of dolphins are mostly based on their foraging behavior for the prey of these cetaceans. Unfortunately, there are no studies about the diet of the common and bottlenose dolphins in the Upper Gulf of California to analyze the spatial–temporal distributions of prey and predators (dolphins). This kind of information is very important to try to understand why the sampling site location variable, together with the night-day condition, was always listed in the formulation of variables of the models with the lower values of AIC (Table 4).

In other studies, bottlenose dolphins have been reported to have a broad diet of pelagic and demersal fishes (Walker, Potter & Macko, 1999; Santos et al., 2007; Hernandez-Milian et al., 2015). The squid of the genus *Lolliguncula* also have been reported as a prey item frequent in the stomach contents of bottlenose dolphins (Barros & Odell, 1990; Pate & McFee, 2012). Prey for common dolphins mainly includes sardine and anchovies fish schools (Young & Cockcroft, 1994; Pusineri et al., 2007; Garcia-Gobos et al., 2007; Santos et al., 2013). While in the Upper Gulf of California there is not a commercial fishing activity of sardine and anchovies, this area is an important spawning ground for anchovies (*Anchoa* spp) (Sánchez-Velasco et al., 2012). The main fish species of commercial importance in the region are the bigeye croaker (*Micropogonias megalops*), Spanish mackerel (*Scomberomorus concolor*), and the curvina (*Cynoscion othonopterus*) (Cudney-Bueno & Turk-Boyer, 1998; Rodríguez-Quiroz et al., 2012). The distribution of Panama brief squid (*Lolliguncula panamensis*) includes all the Gulf of California (Arizmendi-Rodríguez et al., 2012), and potentially could be a prey item for bottlenose dolphins. In stomach contents of vaquitas, the brief squid (*Lolliguncula* spp) and adult species of anchovies have been reported (Vidal, Brownell & Findley, 1999). Moreover, bottlenose and common dolphins are known to forage on different species depending on the availability of the prey (Evans, 1982; Blanco, Salomón & Raga, 2001).

The interaction of dolphins with human activities could also affect the distribution of dolphins and therefore the detection of their echolocation clicks. For example, Castellote et al. (2015) results showed that bottlenose dolphins preferred months of low intensity of recreational activity suggesting that human presence might also play an important role in bottlenose dolphin seasonality. According to Lusseau (2014) the exposure of dolphins to human activities has a seasonal displacement effect in the behavior of these animals. However, in our study we do not suggest that anthropogenic activities could have a potential effect in the echolocation click activity of dolphins since our period study (19th June to 19th August) was within the low fishing season in the Upper Gulf California described by Cudney-Bueno & Turk-Boyer (1998). The acoustic monitoring program was carried out during this season to avoid potential loss of equipment for vandalism or illegal fishing activities inside the Vaquita Refuge. Furthermore, we are clear that boat sonar used to infer the presence of boats in our study, although recorded in all sampling seasons (see Table 2), had the limitation that small artisanal fishing boats commonly do not use an echo-sounder for fishing. To our knowledge, the use of an echo-sounder is only for trawlers, small sportfishing boats, or Mexican Navy boats in our study area.

Furthermore, since the spatial distribution of dolphins, like vaquita, overlaps with legal and illegal gillnet fishing, the chance of bycatch mortality is high. Although enforcement and surveillance efforts have not been effective in avoiding vaquita mortality, ongoing efforts need to be continued since other cetacean populations are facing the same threats. During operations of patrolling and/or removing illegal gillnets in the Upper Gulf of California, the Federal Attorney’s Office for Environmental Protection of Mexico, the organizations Sea Shepherd Conservation Society, and Museo de la Ballena y Ciencias del Mar, have documented the entanglement of at least 3 common dolphins (*Delphinus* spp), 12 long-beaked common dolphins, 2 bottlenose dolphins, 1 baleen whale, and 4 humpback whales (*Megaptera novaeangliae*) in gillnets (mainly for totoaba) during the winter 2015 to winter 2020 (Supplemental Information 5).

Finally, the Biosphere Reserve was created to protect the ecosystems, the biodiversity, and species that are ecologically and commercially important, endemic or at risk of extinction (DOF, 1993). Long-beaked common dolphins and bottlenose dolphins are under the category of special protection in the environmental regulations of Mexico, with goals of protection, conservation and sustainable use (DOF, 2010). Therefore, the results of our study about the distribution of dolphins, which are top predators in the ecosystem, are relevant to improve or strengthen the management decisions for the conservation of vaquitas in the Biosphere Reserve, as well as for better conservation of populations of other cetaceans in the Upper Gulf of California.

## Conclusions

Our results suggest that passive acoustic monitoring of dolphins with C-PODs is an effective method for describing temporal and spatial echolocation click distribution patterns of bottlenose and common dolphins of the Upper Gulf of California.

We found a marked diel pattern in the dolphins’ acoustic activity with a significant increase of activity during the night. The spatial distribution of the echolocation click rates per day predicted by the best GLM model was similar to the distribution of observed data. The higher acoustic activity of the dolphins was distributed in the sampling sites located in the eastern of the Vaquita Refuge. Given the causes of echolocation clicks in dolphins, the most plausible explanation for the areas with higher echolocation activity could be related to foraging activity, mainly during nighttime. Finally, the systematic sampling array and the database of the acoustic monitoring program of vaquita provided an excellent opportunity to establish a solid baseline knowledge for dolphin seasonal occurrence and spatial distribution patterns at the Vaquita Refuge, which forms part of Upper Gulf of California and Colorado River Delta Biosphere Reserve.

##  Supplemental Information

10.7717/peerj.9121/supp-1 Supplemental Information 1 Algorithm developed to estimate the false positive rate of click trains classified as dolphins (other cetaceans)

10.7717/peerj.9121/supp-2 Supplemental Information 2 Effort and dolphin clicks collected per sampling site during the summers of 2011 to 2015 The sampling period for each year was from June 19th to August 19th.

10.7717/peerj.9121/supp-3 Supplemental Information 3 Database of the variables used for general and GLM analysis The database is on a one-hour scale.

10.7717/peerj.9121/supp-4 Supplemental Information 4 Routines in R for exploration of data, statistical tests, and GLM analysis

10.7717/peerj.9121/supp-5 Supplemental Information 5 Records of cetaceans entangled in gillnets in the Vaquita Refuge and in Biosphere Reserve of the Upper Gulf of California and Colorado River Delta The list includes only records of individuals with the fishing gear still entangled in the body or with mutilated fins. Hidalgo PE, Sánchez L. 2020. Encountered dead cetaceans during Operation Milagro efforts. Internal Report. Friday Harbor: Sea Shepherd Conservation Society.

## Additional Information and Declarations

### Field Study Permissions

The following information was supplied relating to field study approvals (i.e., approving body and any reference numbers): The Dirección General de Vida Silvestre, Secretaría de Medio Ambiente y Recursos Naturales approved this study (SGPA/DGVS/06998/11, SGPA/DGVS/07105/12).

### Data Availability

The following information was supplied regarding data availability: Raw data and code are available in the Supplemental Files.
