# Supplementary material for: Distribution of the acoustic occurrence of dolphins during the summers 2011 to 2015 in the Upper Gulf of California, Mexico
Source: PeerJ. 2020 May 20;8:e9121. doi: 10.7717/peerj.9121 (PMC7245336; doi:10.7717/peerj.9121)
Supplement: Supplemental Information 2 — The sampling period for each year was from June 19th to August 19th. [file peerj-08-9121-s002.docx]

**Supplemental Information 2.** **Effort and dolphin clicks collected per sampling site during the summers of 2011 to 2015.** The sampling period for each year was from June 19th to August 19th.

|  | **Sampled days** | |  |  |  | **Dolphin clicks** | |  |  |  |
| --- | --- | --- | --- | --- | --- | --- | --- | --- | --- | --- |
| **Site** | **2011** | **2012** | **2013** | **2014** | **2015** | **2011** | **2012** | **2013** | **2014** | **2015** |
| **1** | 62 | 61 | 58 | 62 | 62 | 45,029 | 18,291 | 21,562 | 91,364 | 33,940 |
| **2** | 41 | 61 | 58 | 62 | 62 | 9,451 | 1,412 | 13,734 | 10,588 | 3,942 |
| **3** | 62 | 61 | X | 62 | 62 | 63,174 | 83 | X | 6,667 | 4,532 |
| **4** | 62 | 61 | 58 | 62 | 62 | 15,812 | 4,237 | 3,752 | 25,724 | 30,820 |
| **5** | 62 | 61 | 58 | 62 | 18 | 24,825 | 4,676 | 174 | 23,224 | 8 |
| **6** | 62 | 61 | 58 | 62 | 62 | 90,855 | 51 | 3,462 | 20,964 | 10,137 |
| **7** | 62 | 61 | 37 | 62 | 62 | 64,554 | 3,622 | 13,232 | 55,149 | 34,248 |
| **8** | X | 61 | 21 | 62 | 62 | X | 4,270 | 4,451 | 61,896 | 16,596 |
| **9** | 62 | 61 | 62 | 62 | 47 | 2,625 | 10,863 | 6,216 | 16,082 | 13,754 |
| **10** | X | 61 | 62 | 62 | 62 | X | 7,152 | 9,996 | 16,314 | 16,452 |
| **11** | 62 | 61 | 37 | 62 | 62 | 39,184 | 7,156 | 5,231 | 11,292 | 4,893 |
| **12** | X | 62 | X | X | 62 | X | 3,061 | X | X | 3,496 |
| **13** | 58 | 61 | 62 | 62 | 62 | 13,905 | 3,373 | 1,907 | 19,970 | 11,117 |
| **14** | 58 | 60 | 62 | 62 | 62 | 10,738 | 87 | 36,710 | 10,926 | 8,769 |
| **15** | 57 | 61 | 62 | X | 22 | 28,584 | 0 | 3,241 | X | 30 |
| **16** | 62 | 61 | 62 | 62 | 62 | 1,927 | 637 | 9,777 | 14,323 | 15,985 |
| **18** | X | X | X | X | 62 | X | X | X | X | 5,075 |
| **19** | 62 | 60 | 10 | 62 | 23 | 11 | 0 | 275 | 1,048 | 118 |
| **20** | 62 | 60 | 29 | 62 | 59 | 2,017 | 0 | 755 | 6,409 | 4,817 |
| **21** | 58 | 60 | 37 | 62 | 62 | 3,895 | 35 | 3,037 | 1,724 | 46 |
| **22** | 62 | 60 | 55 | X | 51 | 6,943 | 2,960 | 1,351 | X | 1,382 |
| **23** | 62 | 60 | 62 | 62 | 62 | 18,197 | 17,794 | 1,227 | 3,477 | 16,077 |
| **24** | X | 60 | 62 | 62 | 62 | X | 2,852 | 3,829 | 1,719 | 11,627 |
| **25** | 49 | 62 | 62 | 62 | 62 | 1,187 | 2,105 | 4,594 | 1,241 | 5,091 |
| **26** | 52 | 59 | 62 | 62 | 62 | 0 | 0 | 65,330 | 194 | 1,516 |
| **27** | 62 | 53 | 62 | X | 62 | 2,793 | 0 | 11,824 | X | 1,573 |
| **28** | 62 | 62 | 62 | 62 | 49 | 0 | 104 | 6,964 | 3,819 | 2,495 |
| **29** | 57 | 62 | 62 | X | 62 | 429 | 694 | 776 | X | 2,845 |
| **30** | 62 | 62 | 62 | 62 | 62 | 1,341 | 986 | 533 | 165 | 289 |
| **31** | 62 | 62 | 42 | 62 | 62 | 2,635 | 0 | 50 | 2,011 | 12,305 |
| **32** | 20 | 62 | 62 | 62 | 62 | 0 | 0 | 0 | 3,872 | 7,943 |
| **34** | X | 62 | 11 | 34 | 62 | X | 840 | 78 | 4,535 | 7,242 |
| **35** | 62 | 62 | 50 | 62 | 39 | 660 | 197 | 285 | 5,354 | 0 |
| **36** | 48 | 62 | 44 | 62 | 62 | 0 | 340 | 332 | 2,058 | 706 |
| **37** | 47 | 62 | 45 | 62 | 62 | 807 | 4,234 | 609 | 970 | 1,865 |
| **38** | 62 | 62 | 62 | 62 | 62 | 51 | 50 | 600 | 1,320 | 672 |
| **39** | 61 | 54 | 62 | 62 | 62 | 99 | 0 | 517 | 457 | 128 |
| **40** | 62 | 62 | 62 | 62 | 34 | 12 | 336 | 726 | 481 | 544 |
| **41** | 54 | 34 | 62 | 62 | 62 | 0 | 94 | 55 | 599 | 1,270 |
| **42** | 46 | 62 | 62 | 62 | 62 | 0 | 0 | 0 | 4,512 | 122 |
| **43** | 62 | 62 | 62 | 62 | 53 | 776 | 707 | 1,275 | 31,296 | 62,544 |
| **44** | 62 | 62 | 62 | 62 | 62 | 3,243 | 50 | 194 | 1,610 | 16 |
| **45** | 62 | 62 | 62 | 62 | 62 | 0 | 0 | 0 | 725 | 185 |
| **46** | 48 | 62 | 49 | 62 | 62 | 0 | 0 | 351 | 1,098 | 1,313 |
| **47** | 57 | 62 | 62 | 62 | 62 | 6,211 | 3,142 | 5,377 | 1,051 | 548 |
| **48** | 43 | 62 | 54 | 62 | 61 | 2,541 | 5,891 | 55 | 427 | 350 |
| **Totals** | **2,280** | **2,714** | **2,299** | **2,452** | **2,626** | **464,511** | **112,382** | **244,444** | **466,655** | **359,423** |
