# Supplementary material for: Distribution of the acoustic occurrence of dolphins during the summers 2011 to 2015 in the Upper Gulf of California, Mexico
Source: PeerJ. 2020 May 20;8:e9121. doi: 10.7717/peerj.9121 (PMC7245336; doi:10.7717/peerj.9121)
Supplement: Supplemental Information 5 — The list includes only records of individuals with the fishing gear still entangled in the body or with mutilated fins. Hidalgo PE, Sánchez L. 2020. Encountered dead cetaceans during Operation Milagro efforts. Internal Report. Friday Harbor: Sea Shepherd Conservation Society. [file peerj-08-9121-s005.docx]

**Supplemental Information 5.**

**Records of cetaceans entangled in gillnets in the Vaquita Refuge and in Biosphere Reserve of the Upper Gulf of California and Colorado River Delta.** The list includes only records of individuals with the fishing gear still entangled in the body or with mutilated fins.

| **Date** | **Common name** | **Number** | **Location and/or geographic position** | **Source and comments** |
| --- | --- | --- | --- | --- |
| 12/28/2015 | Humpback whale | 1 | Vaquita Refuge | PROFEPA (Federal Attorney's Office for Environmental Protection). Entangled in gillnet. |
| 02/17/2016 | Humpback whale | 1 | Vaquita Refuge | PROFEPA. Sea Shepherd Conservation Society (Hidalgo and Sanchez, 2020). Entangled in totoaba gillnet. |
| 03/06/2016 | Common dolphin (Delphinus spp) | 1 | Biosphere Reserve  31° 31.26' N  114° 41.93' W | Sea Shepherd Conservation Society (Hidalgo and Sanchez, 2020). Entangled in totoaba gillnet. |
| 22/12/2016 | Long-beaked common dolphin | 1 | San Felipe*  31° 1.468' N  114° 49.924' W | PROFEPA. Stranding. Mutilated fluke |
| 12/29/2016 | Bottlenose dolphin | 1 | San Felipe  30° 59.70' N  114° 49.246' W | Sea Shepherd Conservation Society (Hidalgo and Sanchez, 2020). Stranding. Entangled in totoaba gillnet. |
| 1/02/2017 | Bottlenose dolphin | 1 | Vaquita Refuge  31°07.6' N  114°42.8' W | Sea Shepherd Conservation Society (Hidalgo and Sanchez, 2020). Mutilated fluke |
| 2/9/2017 | Long-beaked common dolphin | 1 | San Felipe Bay  30°59.225'N  114°45.781' W | Sea Shepherd Conservation Society (Hidalgo and Sanchez, 2020). Dorsal, pectoral, and fluke mutilated. |
| 2/11/2017 | Long-beaked common dolphin | 1 | Biosphere Reserve  31°07.62' N  114°47.24' W | Sea Shepherd Conservation Society (Hidalgo and Sanchez, 2020). Entangled in gillnet. Fluke mutilated |
| 2/13/2017 | Long-beaked common dolphin | 1 | Vaquita Refuge | PROFEPA. Dorsal, pectoral and fluke fin mutilated |
| 2/15/2017 | Common dolphin | 1 | San Felipe  31° 2.136' N  114° 49.604' W | CONANP (GCH). Stranded. Entangled in gillnet. |
| 2/17/2017 | Humpback whale | 1 | Biosphere Reserve | PROFEPA. Entangled in totoaba gillnet. |
| 2/21/2017 | Long-beaked common dolphin | 1 | Biosphere Reserve  31° 15.88' N  114° 47.50' W | Sea Shepherd Conservation Society (Hidalgo and Sanchez, 2020). Entangled in totoaba gillnet. |
| 3/8/2017 | Baleen whale | 1 | Biosphere Reserve  31° 07.15' N  114° 48.014' W | Sea Shepherd Conservation Society (Hidalgo and Sanchez, 2020). Entangled in totoaba gillnet. |
| 3/22/2017 | Long-beaked common dolphin | 1 | Biosphere Reserve  31° 24.15' N  114° 46.69' W | Sea Shepherd Conservation Society (Hidalgo and Sanchez, 2020). Entangled in totoaba gillnet. |
| 02/26/2019 | Long-beaked common dolphin | 1 | Biosphere Reserve  31° 02.832' N  114° 48.788' W | Museo de la Ballena y Ciencias del Mar, A.C. Mutilated fluke. |
| 03/13/2019 | Common dolphin | 1 | Biosphere Reserve  31° 03.416' N  114° 44.654' W | Museo de la Ballena y Ciencias del Mar, A.C. Entangled in gillnet |
| 03/23/2019 | Long-beaked common dolphin | 1 | Biosphere Reserve  31º 05.04' N  114º 46.32' W | Sea Shepherd Conservation Society (Hidalgo and Sanchez, 2020). Fluke mutilated |
| 03/27/2019 | Long-beaked common dolphin | 2 | Vaquita Refuge  31° 07.452 N'  114° 45.587' W | Museo de la Ballena y Ciencias del Mar, A.C. Entangled in gillnet. One with fluke mutilated, Other with scars of nets |
| 11/15/2019 | Long-beaked common dolphin | 1 | Vaquita Refuge  31º 02.80' N  114º 25.87' W | Sea Shepherd Conservation Society (Hidalgo and Sanchez, 2020). Entangled in totoaba gillnet. |
| 01/26/2020 | Long-beaked common dolphin | 1 | Vaquita Refuge  31º 04.332' N  114º 41.359' W | Sea Shepherd Conservation Society (Hidalgo and Sanchez, 2020). Entangled in totoaba gillnet. |
| 02/23/2020 | Humpback whale | 1 | Vaquita Refuge | PROFEPA. Sea Shepherd Conservation Society (Hidalgo and Sanchez, 2020). Entangled in totoaba gillnet. |

* San Felipe is not located in the Biosphere Reserve of the Upper Gulf of California, but the small town is located at the same latitude as Vaquita Refuge (Fig. 1).
